# Chaotropic and Kosmotropic Anions Regulate the Outcome of Enzyme-Mediated Dynamic Combinatorial Libraries of Cyclodextrins in Two Different Ways

**DOI:** 10.3389/fchem.2021.721942

**Published:** 2021-08-03

**Authors:** Andreas Erichsen, Dennis Larsen, Sophie R. Beeren

**Affiliations:** Department of Chemistry, Technical University of Denmark, Kongens Lyngby, Denmark

**Keywords:** dynamic combinatorial chemistry, supramolecular chemistry, anions, cyclodextrins, cyclodextrin glucanotransferase, templated synthesis, systems chemistry

## Abstract

We demonstrate how different anions from across the Hofmeister series can influence the behavior of enzyme-mediated dynamic combinatorial libraries of cyclodextrins (CDs). Using cyclodextrin glucanotransferase to catalyze reversible transglycosylation, dynamic mixtures of interconverting cyclodextrins can be formed wherein the relative concentrations of α-CD, β-CD and γ-CD is determined by their intrinsic stabilities and any stabilizing influences of added template (guest) molecules. Here, we find that addition of high concentrations of kosmotropic anions can be used to enhance the effects of added hydrophobic templates, while chaotropic anions can themselves act as templates, causing predictable and significant changes in the cyclodextrin composition due to weak, but specific, binding interactions with α-CD.

## Introduction

Since the groundbreaking work of Franz Hofmeister more than a century ago into how salts affect the solubility of proteins (Hofmeister, 1888), countless studies have repeatedly revealed the Hofmeister series of anions: F^−^, SO_4_^2−^, AcO^−^, Cl^−^, Br^−^, NO_3_^−^, ClO_3_^−^, I^−^, ClO_4_^−^_,_ and SCN^−^. Kosmotropes, such as F^−^ and SO_4_^2−^, generally decrease the solubility of proteins and other solutes (salting out), and while the rules governing these phenomena are not fully understood, these ions are said to enhance the hydrophobic effect (Gibb, 2011). Chaotropes, such as ClO_4_^−^ and SCN^−^, generally increase the solubility of proteins and other solutes (salting in), cause the denaturation of proteins at high concentrations and diminish the hydrophobic effect. The affinity of chaotropic anions towards hydrophobic surfaces allow them to compete with the interactions between hydrophobic solutes (Gibb and Gibb, 2011). Chaotropic anions form complexes with hosts that have hydrophobic cavities, such as cavitands and cyclodextrins (CDs) (Sullivan et al., 2018). The binding of chaotropic anions to hydrophobic solutes is associated with a certain thermodynamic fingerprint—a favorable enthalpy and an entropic penalty—which has recently been characterized as a generic driving force under the term “the chaotropic effect” by Nau and coworkers (Assaf et al., 2015; Assaf and Nau, 2018). These authors stressed that the chaotropic effect should be distinguished from the classical hydrophobic effect, where the thermodynamic signature is a favorable entropic term. Understanding the different influences of kosmotropes, chaotropes and hydrophobes on self-assembly processes in aqueous solution, both as modulators of solvent effects (Kunz et al., 2004; Gibb, 2011, Gibb, 2019; van der Vegt and Nayar, 2017) and as recognition motifs (Busschaert et al., 2015; Hillyer and Gibb, 2016; Assaf and Nau, 2018), is key to the successful design of supramolecular systems. In this work, we examine how the interplay between kosmotropic, chaotropic and hydrophobic effects modulates the behavior of an enzyme-mediated dynamic system of cyclodextrins.

Dynamic combinatorial chemistry (DCC) is a powerful method to explore molecular self-assembly and the templated synthesis of complex molecular architectures using reversible bond formation under thermodynamic control (Otto et al., 2002; Corbett et al., 2006; Lehn, 2007; Ponnuswamy et al., 2012; Li et al., 2013). We have previously described how dynamic combinatorial libraries (DCLs) of interconverting cyclodextrins can be generated by employing an enzyme that enables reversible transglycosylation (Larsen and Beeren, 2019, Larsen and Beeren, 2020, Larsen and Beeren, 2021). Cyclodextrins are macrocycles formed from α-1,4-linked glucopyranose units. The native cyclodextrins, α-CD, β-CD and γ-CD, with six, seven, and eight glucopyranose units, respectively, exhibit truncated cone-like structures and are widely utilized hosts for the encapsulation of hydrophobic molecules in the foods, pharmaceutical and cosmetics industries (Hedges, 1998; Del Valle, 2004; Sharma and Baldi, 2016). Cyclodextrin glucanotransferase (CGTase) catalyzes both fast, reversible inter- and intramolecular transglycosylation and slow hydrolysis of α (1–4)-glycosidic bonds (Terada et al., 1997; Tewari et al., 1997; Uitdehaag et al., 2002). Exposure of an α-1,4-glucan source to CGTase, therefore, generates a dynamic mixture of linear α-1,4-glucans (maltooligosaccharides) and cyclic α-1,4-glucans (cyclodextrins) (Larsen and Beeren, 2019). As α-CD, β-CD and γ-CD are intrinsically more stable than their linear counterparts, a complex dynamic system is formed in which α-CD, β-CD and γ-CD are kinetically trapped and transiently form as the primary products before being eventually converted to glucose.

**FIGURE 1:**
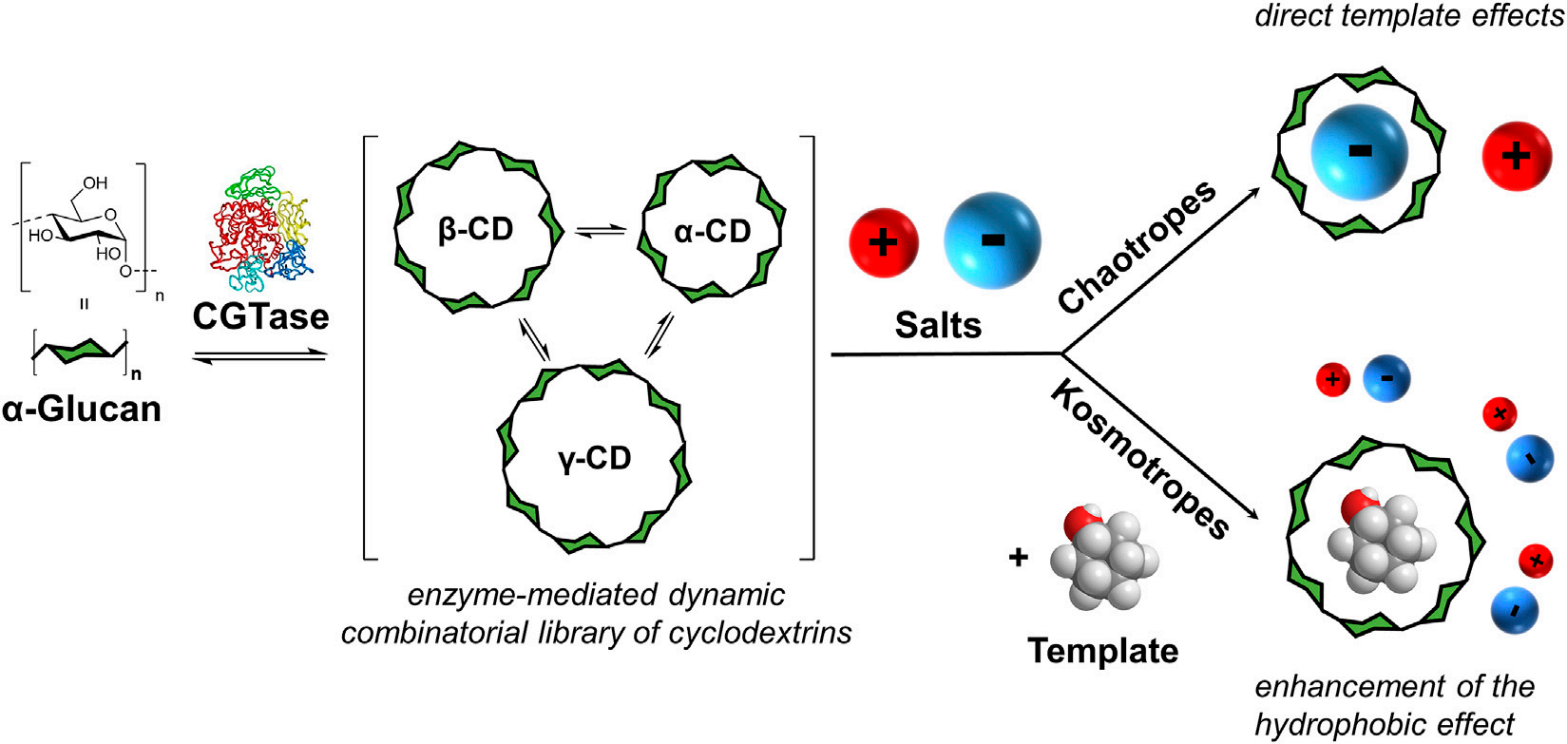
Concept of enzyme-mediated dynamic combinatorial chemistry with α-1,4-glucans and the effect of added salts. Cyclodextrin glucanotransferase (CGTase) acts on α-1,4-glucans to generate a dynamic mixture of cyclodextrins. Addition of salts leads to either direct template effects (with chaotropic salts) or to the enhancement of the template effect of an added guest (with kosmotropic salts).

We previously showed that even though α-CD, β-CD and γ-CD form out-of-equilibrium in this enzyme-mediated dynamic system, they exist in a subsystem that operates under *pseudo*-thermodynamic control (Larsen and Beeren, 2019). The distribution of products formed can be controlled by addition of a template that binds selectively to specific cyclodextrins. We were able to produce α-CD, β-CD or γ-CD with 99% selectivity using sodium dodecyl sulfate, 1-adamantanecarboxylic acid and sodium tetraphenylborate as templates (Larsen and Beeren, 2019, Larsen and Beeren, 2020). While investigating different reaction conditions, it was also found that CGTase could not only tolerate very high concentrations of NaNO_3_ (up to 7.5 M) but that the presence of NaNO_3_ altered the distribution of cyclodextrins in the DCL that was formed. These results encouraged us to explore how the addition of a range of sodium salts in high concentrations would influence the cyclodextrin distribution in our dynamic system. Here, we present how the addition of salts to CGTase-mediated DCLs of cyclodextrins can either enhance the template effects of added guests, in the case of kosmotropes, or lead to direct template effects, in the case of chaotropes (Figure 1).

## Materials, Instrumentation and Methods

### Materials

All chemicals and solvents of HPLC (high performance liquid chromatography) grade were obtained from commercial suppliers and used as received. High purity water used both in reactions and chromatographic analysis was obtained using a Merck Millipore Synergy UV water purification system. Colorless Corning CoStar 0.65 ml centrifuge tubes were used for enzymatic reactions and sample preparation (dilution and centrifugation), while colorless 2 ml glass vials with PTFE-lined screw-cap septa and 0.2 ml glass inserts were used for short-term sample storage and injection on HPLC equipment. A stock solution of the enzyme CGTase derived from *Bacillus macerans* was received as a kind gift from Amano Enzyme, Inc., Nagoya, Japan. The stock solution was stored at 5°C and used as received.

### Instrumentation

Chromatographic analysis was performed on a Thermo Scientific Dionex Ultimate 3000 HPLC (ultra-high pressure) system equipped with a Waters Acquity UPLC BEH Amide 1.7 µm 2.1 × 150 mm column maintained at 30°C, and an autosampler module maintained at 20°C. Detection was carried out using an Agilent Technologies 1,260 Infinity ELSD (evaporative light scattering detector), operating with the evaporator at 90°C, nebulizer at 70°C, and a N_2_ gas flow of 1.0 L/min. The ELSD enables the detection of the chromophore-lacking oligosaccharides. Calibration curves from 0.01 mg/ml to 10 mg/ml for α-, β- and γ-CD and linear α-1,4-glucans up to maltooctaose were used to correct for differences in the ELS detector response for different oligosaccharides. The calibrations were based on masses injected (0.018–3.66 µg) and the resulting response curves were fitted to a simple power equation *M* = *kA* ^*p*^ (where *M* is the injected mass of compound, *A* is the area under the peak in the chromatogram and *k* and *p* are fitted parameters) using non-linear curve fitting (in OriginPro 2018b from OriginLab Corp.) See recent paper for details (Larsen and Beeren, 2021). The gradient profile for HPLC runs was a linear gradient from 75% acetonitrile in water to 55% acetonitrile in water over 8 min with a flow rate of 0.6 ml/min. Both eluents contained 0.1% formic acid by volume.

### Enzymatic Reactions and Analysis

Reaction mixtures with the desired concentrations of salts were prepared by mixing appropriate amounts of two types of stock solutions in buffered water (50 mM sodium phosphate at pH 7.5): 1) a solution containing α-CD (10 mg/ml) and various salts (4.0 M); and 2) a solution containing α-CD (10 mg/ml). In templated experiments, cyclohexanol or cyclohexanecarboxylic acid was dissolved in these resulting mixtures at a concentration of 10 mM. For the experiments with a higher concentration of sodium phosphate buffer, a stock solution of α-CD (10 mg/ml) in 0.45 M sodium phosphate buffer at pH 7.5 was prepared. The starting mixtures containing salt, α-CD and template (if any) in buffer were then aliquoted (165–365 µl) into reaction vessels and kept at ambient temperature. All reactions were then initiated by adding CGTase stock solution (50 µl per ml of starting mixture) to the starting mixtures followed by thorough mixing. The reactions were then monitored at various time points: Aliquots for analysis (4–5 µl) were taken out and rapidly diluted (31 fold) in a 1% trifluoroacetic acid (TFA) solution in 3:1 acetonitrile/water with 10 mM ammonium chloride to stop the enzymatic reaction. For experiments with NaCl, aliquots for analysis (20 µl) were taken out and rapidly diluted (six fold) in a 1% TFA solution in water. Samples were then centrifuged (10,000 RPM for 4 min) to prevent column blockage by insoluble species such as enzyme and salts, and the top fractions (leaving behind 20 µl) were then transferred to 2 ml glass vials with 0.2 ml glass inserts, kept at 20°C and then injected on the HPLC instrument within 48 h. Injection volumes were 10 µl or 2 µl (for 31-fold and 6-fold diluted samples, respectively). Peaks in the chromatograms corresponding to α-CD, β-CD and γ-CD and linear α-1,4-glucans up to maltooctaose were identified by comparison with authentic samples obtained from commercial suppliers.

### Simulations of Dynamic Combinatorial Libraries

Simulations of dynamic combinatorial libraries (DCLs) with and without the anion SCN^−^ were carried out using the *DCLSim* software developed in the Otto group (Corbett et al., 2004) and kindly made available to us. The program requires input of the concentration of the building block (glucopyranose units in this case), the composition of the oligomers (library members) formed in the DCL (α-CD, β-CD and γ-CD, with six, seven or eight glucose units in this case), the relative formation constants *K*_f_ of the library members (determined from the *pseudo*-equilibrium composition of α-, β-, and γ-CD in an untemplated library), the binding constants (*K*_a_) of each library member to the template (SCN^−^) and the concentration of the template. Details about how the relative formation constants *K*_f_ of α-, β-, and γ-CD were calculated can be found in the Supplementary Material. The binding constants used were obtained from a study carried out by Tokunaga and coworkers, where the authors used ^1^H-NMR spectroscopy to determine binding constants between inorganic anions and cyclodextrins (Matsui et al., 1997).

## Results and Discussion

### The Influence of a Series of Anions on Cyclodextrin DCLs

To explore the influence of anions on CGTase-mediated DCLs of cyclodextrins, we examined a series of DCLs prepared in the presence of different sodium salts at concentrations up to 4 M. The following series of anions was investigated, ranked according to the Hofmeister series: HPO_4_^2−^/H_2_PO_4_^−^ > Cl^−^ > NO_3_^−^ > Br^−^ > ClO_4_^−^ > SCN^−^. The libraries were prepared by addition of CGTase (50 μl stock solution per ml reaction mixture) to solutions of α-CD (10 mg/ml) with the desired sodium salts at various concentrations up to 4 M concentration in phosphate buffer (50 mM, pH 7.5). Despite the lower solubility of sodium phosphates, we chose also to include phosphate buffer in this study albeit at a maximum of 0.45 M. The library compositions were monitored as the dynamic system evolved over time using hydrophilic interaction liquid chromatography (HILIC) with an evaporative light scattering detector (ELSD), which enabled the separation and quantification of the chromophore-lacking glucan mixtures. The influence of varying concentrations of different salts on the equilibrium cyclodextrin distribution and the time taken to reach this steady distribution is summarized for all anions tested in Table 1.

**TABLE 1:** Summary of results (relative CD yield at *pseudo*-equilibrium and time to *pseudo*-equilibrium) for CGTase-mediated Dynamic Combinatorial Libraries (DCLs) of cyclodextrins in the presence of different sodium salts.

Entry #	Salt^a^	Salt concentration (M)	Time to *pseudo-*equilibrium (h)^b^	Relative CD yield at *pseudo-*equilibrium (% by weight)^c^		
				α-CD	β-CD	γ-CD
1	No salt	—	1	32	57	11
2	HPO_4_^2-^/H_2_PO_4_^−^	0.45	1	36	54	10
3	NaCl	1	1	37	54	10
4	NaCl	2	2	37	54	9
5	NaCl	3	2	37	53	9
6	NaCl	4	4	40	52	8
7	NaNO_3_	1	1	48	45	7
8	NaNO_3_	2	1.5	49	45	6
9	NaNO_3_	3	2	51	43	6
10	NaNO_3_	4	2	55	40	5
11	NaBr	1	1	41	50	8
12	NaBr	2	1.5	44	49	7
13	NaBr	3	2.5	48	46	6
14	NaBr	4	4	54	40	5
15	NaClO_4_	1	3	62	36	2
16	NaClO_4_	2	6	65	34	1
17	NaClO_4_	3	^d^	—	—	—
18	NaSCN	1	2	72	26	2
19	NaSCN	2	8	75	23	2
20	NaSCN	3	^d^	—	—	—

a Conditions: α-CD (10 mg/ml) in sodium phosphate buffer (50 mM, pH 7.5) treated with CGTase at room temperature.

b Estimated time (to the nearest half hour) until a steady distribution of CDs was obtained.

c Values taken from a single data point after a steady distribution of CDs was obtained. (Instrumental uncertainty of about ±2% points in the relative CD yields).

d Zero or close to zero enzyme activity, presumably due to enzyme denaturation.

**FIGURE 2:**
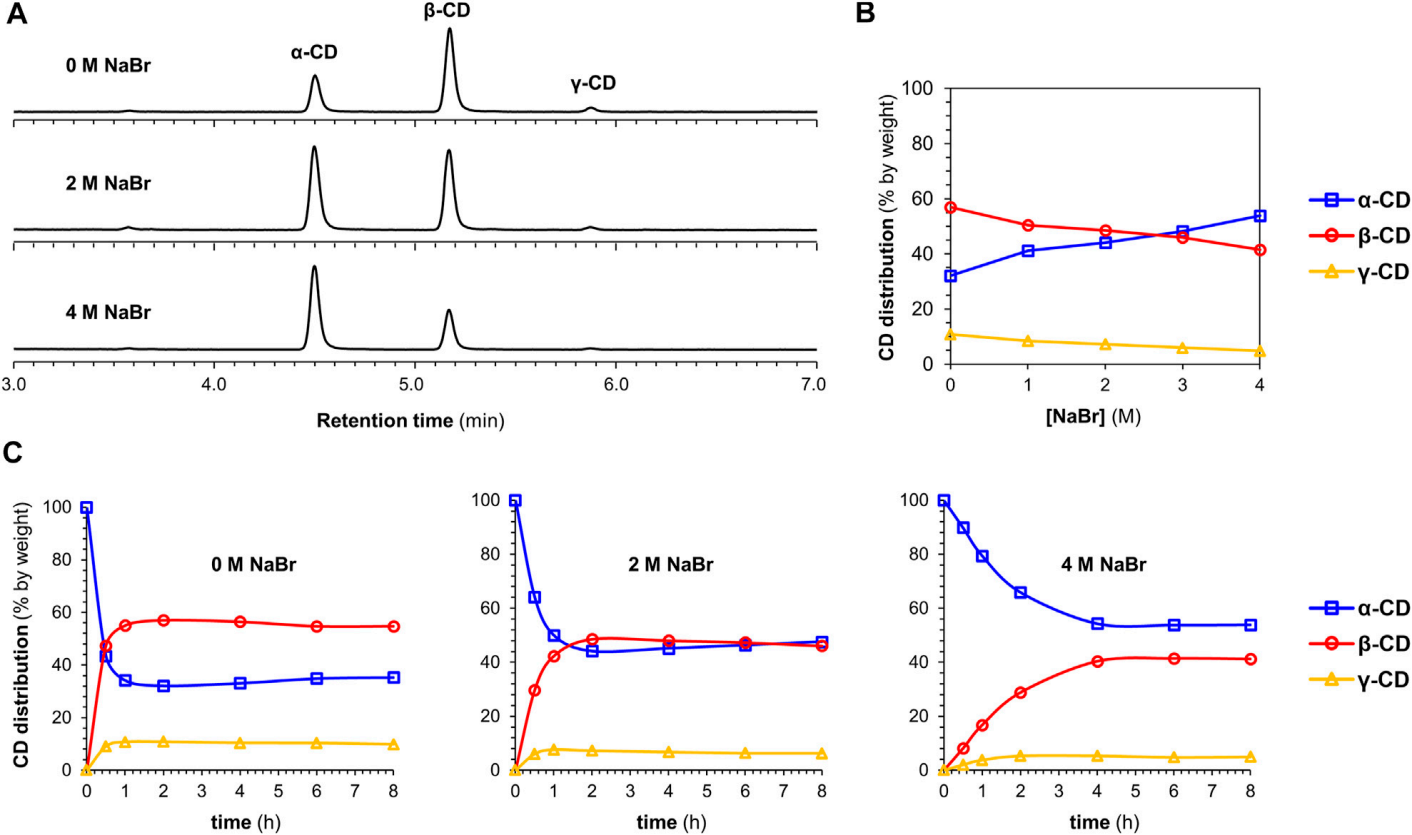
The effect of NaBr on CGTase-mediated DCLs. Conditions: α-CD (10 mg/ml) in sodium phosphate buffer (50 mM, pH 7.5) treated with CGTase at room temperature **(A)** Chromatograms (HPLC-ELS) showing the *pseudo*-equilibrium distribution of CDs produced in the absence or presence of NaBr (2 and 4 M). The chromatograms show data obtained after 2 h (0 and 2 M NaBr) or 6 h (4 M NaBr) **(B)** *Pseudo*-equilibrium distribution of CDs as a function of NaBr concentration **(C)** CD distribution monitored over time in the absence or presence of NaBr (2 and 4 M) (lines are included only to guide the eye).

Figure 2 shows representative data obtained for DCLs of cyclodextrins prepared in the presence of increasing amounts of NaBr. Figure 2A depicts chromatograms showing the distributions of α-1,4-glucan products formed after *pseudo*-equilibrium is obtained (2–6 h) in the absence and presence of different concentrations of NaBr. It is immediately evident that the relative concentration of α-CD increased at the expense of β-CD and γ-CD in the presence of increasing concentrations of NaBr. Amplifications of α-CD were seen for all the anions tested, but the magnitude of the effect was anion-dependent (Table 1). The evolution of each DCL was monitored over time (Supplementary Figures 1–6). For all DCLs, the CD yield decreased gradually overtime, due to background hydrolysis and the consequent build-up of short linear α-1,4-glucan and glucose. For the DCL without salt, a steady distribution of α-CD, β-CD and γ-CD was obtained after approximately one hour, when a *pseudo-*thermodynamic equilibrium of cyclodextrins was reached. At this point >90% of the glucan material was still present as cyclodextrins. With increasing concentrations of NaBr, the evolution of the DCL became slower, and it took up to approximately four hours (with 4 M NaBr) to reach a steady distribution of cyclodextrins (Figure 2C). In all cases, addition of anions led to a slower evolution of the dynamic enzymatic system (Supplementary Figures 1–6). With the chaotropic anions ClO_4_^−^ and SCN^−^ (Table 1, entries 15–20) this retardation was quite significant at 1–2 M concentrations, and at concentrations of 3 M and higher, the activity of the enzyme was zero or close to zero within just 30 min of being exposed to the salt solutions (Supplementary Figure 8), presumably due to denaturation of the enzyme under these conditions.

**FIGURE 3:**
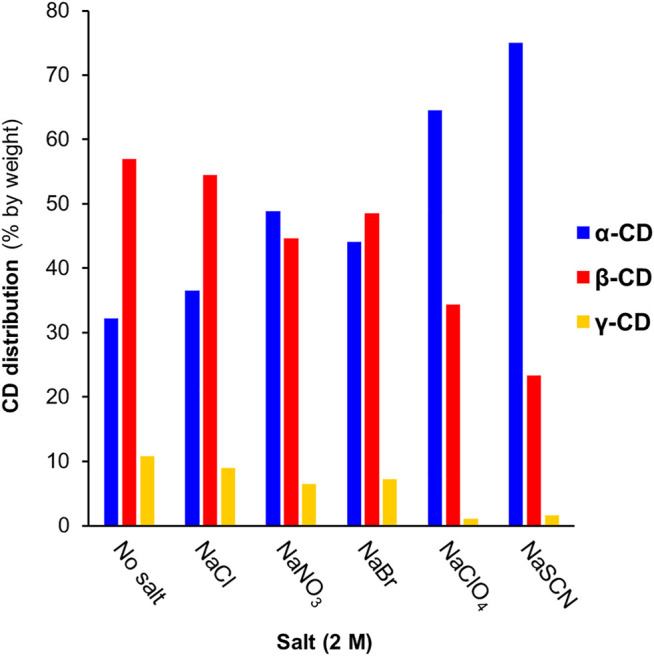
The effect of different anions (at 2 M concentration) on *pseudo-*equilibrium CD distributions obtained in CGTase-mediated DCLs. Conditions: α-CD (10 mg/ml) in sodium phosphate buffer (50 mM, pH 7.5) treated with CGTase at room temperature.

The equilibrium cyclodextrin distributions obtained in the presence of different anions at 2 M concentration are summarized in Figure 3. Following a largely systematic trend, the relative yield of α-CD increases upon moving from the most kosmotropic anions (HPO_4_^2−^/H_2_PO_4_^−^,Cl^−^, minor changes) to the most chaotropic anions (ClO_4_^−^, SCN^−^, large changes). These results suggest that all the tested anions have a specific, albeit weak affinity for α-CD and function as templates in the enzyme-mediated DCL of cyclodextrins. Selected binding constants for Cl^−^, NO_3_^−^, Br^−^, SCN^−^_,_ and ClO_4_^−^ interacting with α-CD, β-CD and γ-CD have previously been reported (Table 2). These binding constants were determined using a variety of techniques: ^1^H-NMR spectroscopy (Matsui et al., 1997), conductance (Wojcik and Rohrbach, 1975), potentiometry (Gelb et al., 1983), spectrophotometry (Buvári and Barcza, 1979), volatilization (Sanemasa et al., 1988) and isothermal titration calorimetry (Sullivan et al., 2018). While the numeric values of the binding constants vary somewhat depending on the method, the trends are certainly clear. In the kosmotropic end of the Hofmeister series, the binding of Cl^−^ to α-CD was found to be negligible in most cases, which corresponds well with our data, where only minor changes in the cyclodextrin distribution occur upon addition of Cl^−^ (Table 1, entries 3–6). The minor increase in the relative yield of α-CD, from 32 to 37%, does, however, indicate that there could be a very weak binding between α-CD and Cl^−^, as found in some cases in the literature (Wojcik and Rohrbach, 1975). Next in the series, both NO_3_^−^ and Br^−^ bind to α-CD with small and similar binding constants (1–4 M^−1^), which matches the small but significant amplification of α-CD observed with these anions (Table 1, entries 7–14). The chaotropic anions SCN^−^ and ClO_4_^−^ have significantly higher affinities for α-CD (16–46 M^−1^) and led to much larger changes in the cyclodextrin distribution (Table 1, entries 15–20). The fact that the amplification of α-CD is larger with SCN^−^ than ClO_4_^−^ can be explained by the relatively higher competing affinity of ClO_4_^−^ for β-CD, as the distribution obtained in a DCL is influenced by the binding interaction of the template with all members of a library. It is noteworthy that each of the small anions tested amplified and bound most strongly to α-CD, whereas we have previously observed the amplification of larger CDs with 8, 9, and 10 glucopyranose units in the presence of the large superchaotropic anion B_12_I_12_^2−^ (diameter 11.7 Å) (Larsen and Beeren, 2019). There is clearly, thus, a relationship between the size of the anion (Table 2 column 3) and the preferential formation of the CD(s) with a suitable size cavity. Overall, we found that the addition of anions in high concentrations to the CGTase-mediated DCLs of cyclodextrins leads to changes characteristic of template effects, and remarkably, the system remains dynamic even at 2 M concentrations of the denaturing salts NaSCN and NaClO_4_.

**TABLE 2:** Binding constants (*K*_*a*_) for anions with α-CD, β-CD and γ-CD reported by various authors and the size (diameter, *d*) of the anions.

Host	Guest	*d* (Å)^c^	*K*_*a*_ (M^−1^)^a^					
			I	II	III	IV	V	VI
α-CD	Cl^−^	3.6	—	<1	—	∼0	3	No binding
	NO_3_^−^	4.0	1.4	1.4	2.31	—	4	^b^
	Br^−^	3.9	1.6	3.5	0.96	—	—	—
	SCN^−^	4.3	28.4	18.7	33.5	—	—	16
	ClO_4_^−^	4.8	33.0	28.9	45.8	—	35	23
β-CD	NO_3_^−^	—	—	—	—	0.2	—	—
	Br^−^	—	—	—	0.45	1.1	—	—
	SCN^−^	—	9.2	9.9	9.2	5.7	—	—
	ClO_4_^−^	—	13.6	—	—	9.0	—	—
γ-CD	SCN^−^	—	4.1	—	—	—	—	—

a Binding constants for anions (as Na^+^ or K^+^ salts) to α, β and γ-CD measured in H_2_O or D_2_O at 20°C or 25°C with various techniques: I) ^1^H-NMR spectroscopy (Matsui et al., 1997); II) Conductance (Wojcik and Rohrbach, 1975); III) Potentiometry (Gelb et al., 1983); IV) Spectrophotometry (Buvári and Barcza, 1979); V) Volatilization (Sanemasa et al., 1988); VI) Isothermal titration calorimetry (Sullivan et al., 2018).

b Binding too weak to determine a binding constant.

c From reference (Marcus, 1994, 1997).

### DCL Simulation to Support Templating Effects of Chaotropes

**FIGURE 4:**
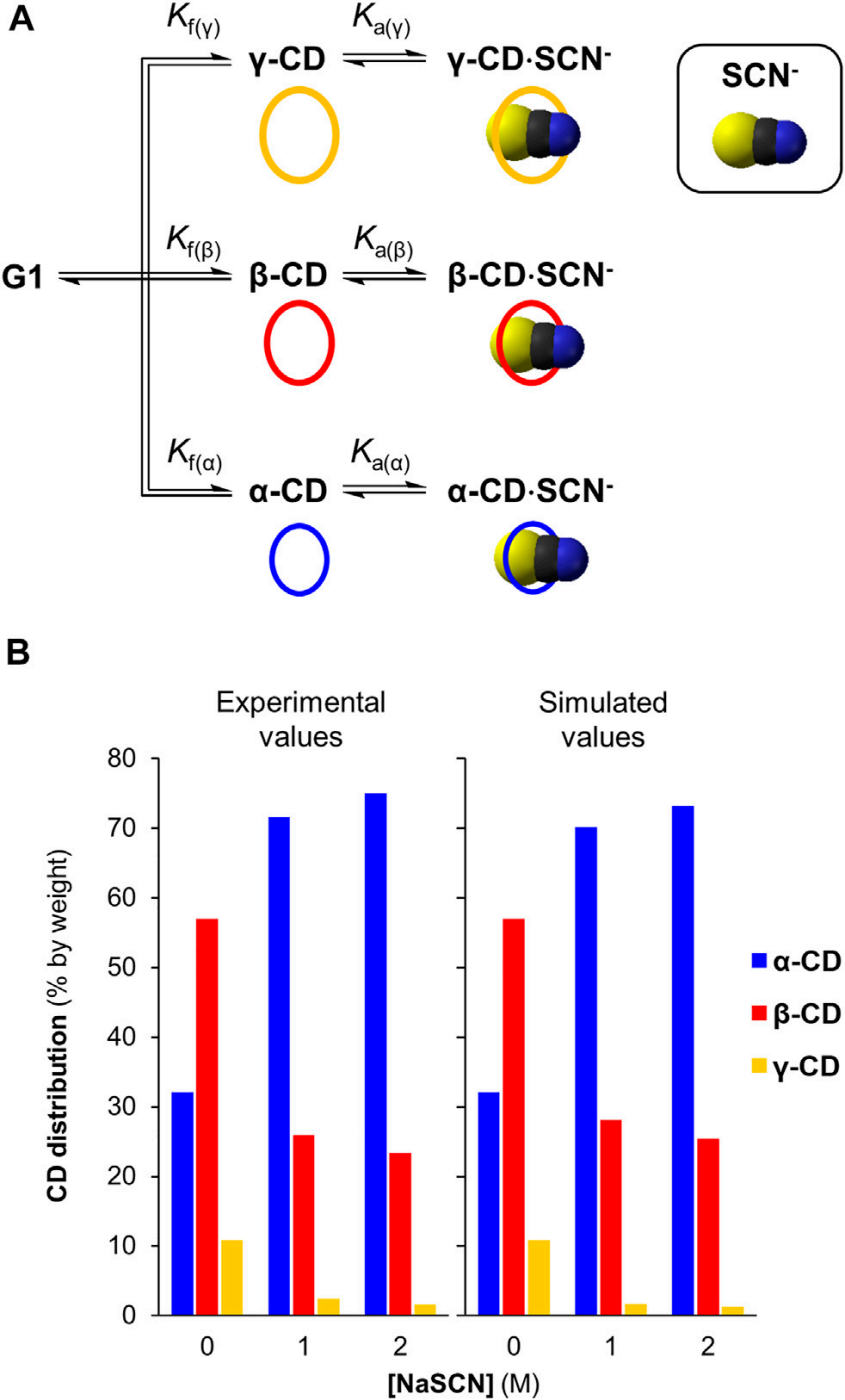
*DCLSim* simulations of DCLs of cyclodextrins with and without the NaSCN **(A)** Model employed to simulate DCLs of α-, β-, and γ-CD in the presence of NaSCN **(B)** Comparison between the simulated and experimentally determined CD distributions with NaSCN (0–2 M).

To gain further support for our conclusion that chaotropic anions influence the production of specific cyclodextrins in CGTase-mediated dynamic systems through direct template effects, we sought to simulate the DCL generated in the presence of increasing concentrations of NaSCN. *DCLSim* is a software developed in the Otto group (Corbett et al., 2004) that enables the prediction of product distributions in templated DCLs operating under thermodynamic control. To simulate the DCLs, binding constants for the interaction of the template with each library member (α-CD, β-CD and γ-CD) is required, and this was available for SCN^−^ (Table 2, column I, Matsui et al., 1997). The relative formation constants *K*_f_ for α-CD, β-CD and γ-CD are also needed for the simulation and these could be calculated from the relative concentrations of α-CD, β-CD and γ-CD generated at equilibrium in the DCL without salt. We simulated DCLs of cyclodextrins with SCN^−^ at 0, 1 and 2 M concentrations. The results, summarized in Figure 4, show that the simulations correlate well with the experimental results, supporting the conclusion that chaotropic anions function as templates in this system, and at high concentrations can strongly influence the product selectivity in CGTase-mediated cyclodextrin synthesis.

### Increasing the Template Effect of Hydrophobic Guests by Addition of a Kosmotropic Salt

**FIGURE 5:**
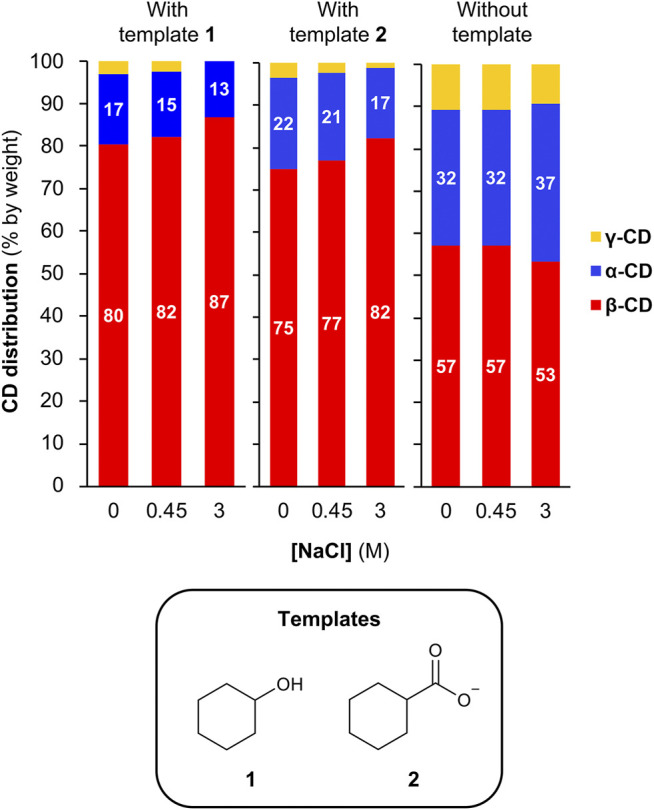
Enhancing the template effect of cyclohexanol and cyclohexane carboxylate by the addition of NaCl. CD distributions from DCLs generated in the absence or presence of template (10 mM) and in the absence or presence of NaCl (up to 3 M). Conditions: α-CD (10 mg/ml) in sodium phosphate buffer (50 mM, pH 7.5) treated with CGTase at room temperature.

As kosmotropic salts can lead to stronger binding between cyclodextrins and hydrophobic guests (Buvári and Barcza, 1979; Holm et al., 2014), we sought to investigate whether we could enhance the template effect of hydrophobic templates in this dynamic cyclodextrin system by using high concentrations of NaCl. For these experiments, we chose to employ cyclohexanol **(template 1)** and cyclohexanecarboxylate **(template 2)** as templates, as both guests have a relatively low affinity for β-CD in the absence of salts (ca. 700 and ca. 300 M^−1^, respectively) (Gelb and Schwartz, 1989; Rekharsky et al., 1994), thus giving room for a possible improvement in templating effect upon addition of NaCl. A series of CGTase-mediated DCLs were set-up starting from α-CD (10 mg/ml) with template (10 mM) in phosphate buffer (50 mM, pH 7.5) with 0–3 M NaCl (Supplementary Figure 7). It was found that the addition of NaCl in increasing concentrations up to 3 M did in fact lead to a moderate increase in the selectivity for β-CD obtained in the presence of each template (from 80 to 87% for template 1 and from 75 to 82% for template 2) (Figure 5). It is worth noting that increasing the concentration of NaCl from 0 to 3 M in the absence of template also changes the cyclodextrin distribution, but in the opposite direction, with a decrease in the relative yield of β-CD from 57 to 53%, thus counteracting the observed increase in the template effect with 1 and 2. Accordingly, the actual kosmotrope-induced increase in the template effect is potentially larger than the effect observed here.

## Conclusion

In this work, we have systematically explored the influence of different anions on the behavior of CGTase-mediated dynamic combinatorial libraries of cyclodextrins and distinguished two key effects. One the one hand, we observed that NaCl (which lies towards the kosmotropic end of the Hofmeister series) could subtly enhance the templating effects of hydrophobic guest added to the DCLs. On the other hand, we observed direct templating effects due to specific interactions between chaotropes and cyclodextrins. These interactions are very weak (*K*_a_ < 50 M^−1^), but, when present in high concentrations (2–4 M), chaotropes can, nevertheless, cause significant changes in the distribution of α-CD, β-CD and γ-CD generated. For example, addition of 2 M NaSCN led to a shift in selectivity from 32% α-CD to 75% α-CD. The observed amplifications of α-CD correlated well with reported binding constants, and simulation of DCLs templated with NaSCN matched well to the experimental results, which is further evidence that the observed effects are due to specific anion-cyclodextrin binding interactions rather than the global influence of the high salt concentration on the bulk solvent. In fact, this dynamic system potentially provides a new method to identify very weak binding of guests to CDs, which would be very difficult to detect otherwise. Finally, we note that the CGTase used in these experiments was remarkably stable at high salt concentrations. Our study showcases how the interplay between kosmotropes, chaotropes and hydrophobes in dynamic supramolecular systems can be utilized to alter the outcome of these systems in predictable and systematic ways.

## Data Availability

The original contributions presented in the study are included in the article/Supplementary Material, further inquiries can be directed to the corresponding author.
