# Supplementary material for: Chaotropic and Kosmotropic Anions Regulate the Outcome of Enzyme-Mediated Dynamic Combinatorial Libraries of Cyclodextrins in Two Different Ways
Source: Front Chem. 2021 Aug 3;9:721942. doi: 10.3389/fchem.2021.721942 (PMC8370642; doi:10.3389/fchem.2021.721942)
Supplement: Supplementary file 1 [file DataSheet1.pdf]

Supplementary Material

1. Enzyme-mediated dynamic combinatorial libraries 2

2. Enzyme activity in the presence of denaturing salts NaSCN and $NaClO_4$ 8

3. Simulations of dynamic combinatorial libraries 9

1 Enzyme-mediated Dynamic Combinatorial Libraries

A series of reactions were set up by treating α-CD (10 mg/mL) with CGTase at room temperature in sodium phosphate buffer in the presence of different sodium salts at concentrations up to 4 M and in the absence or presence of cyclohexanol or cyclohexane carboxylate. The reactions were monitored by HPLC with an ELS detector and the distributions of α-CD, β-CD and γ-CD as a function of time are plotted in Supplementary Figures 1 – 7.

1.1 Sodium phosphate buffer

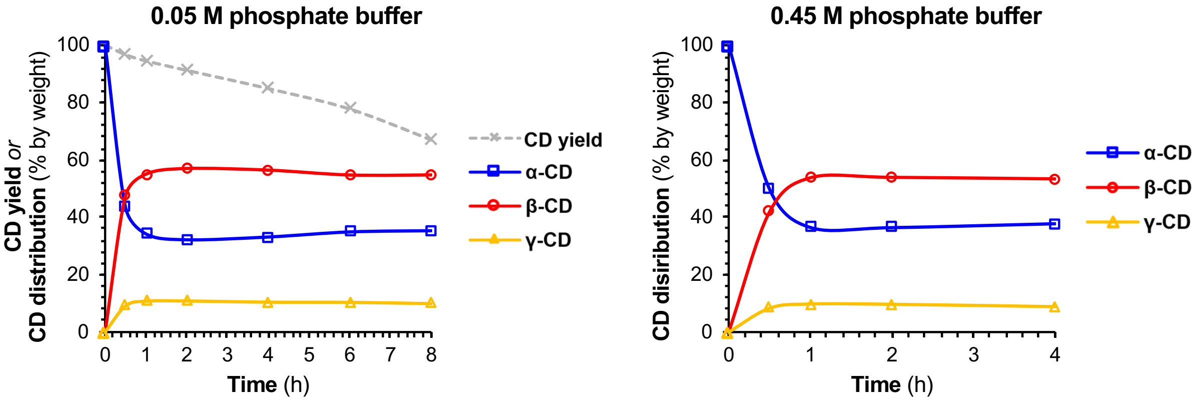

Supplementary Figure 1. Distribution of α-CD, β-CD and γ-CD as a function of time in reactions started from α-CD (10 mg/mL) in sodium phosphate buffer (50 mM or 450 mM, as indicated on graph) at pH 7.5 treated with CGTase at room temperature. (Lines connecting data points are only to guide the eye.)

Note that the total CD yield (grey line, Supplementary Figure 1, left) decreases overtime, due to background hydrolysis and the build-up of short linear α-1,4-glucan and glucose. The same was observed for the DCLs prepared in the presence of salt. However, quantification was not possible as the salt peaks obscured relevant glucan peaks in the chromatograms. In the subsequent figures, only the changes in the distribution of CDs are plotted.

1.2 NaCl

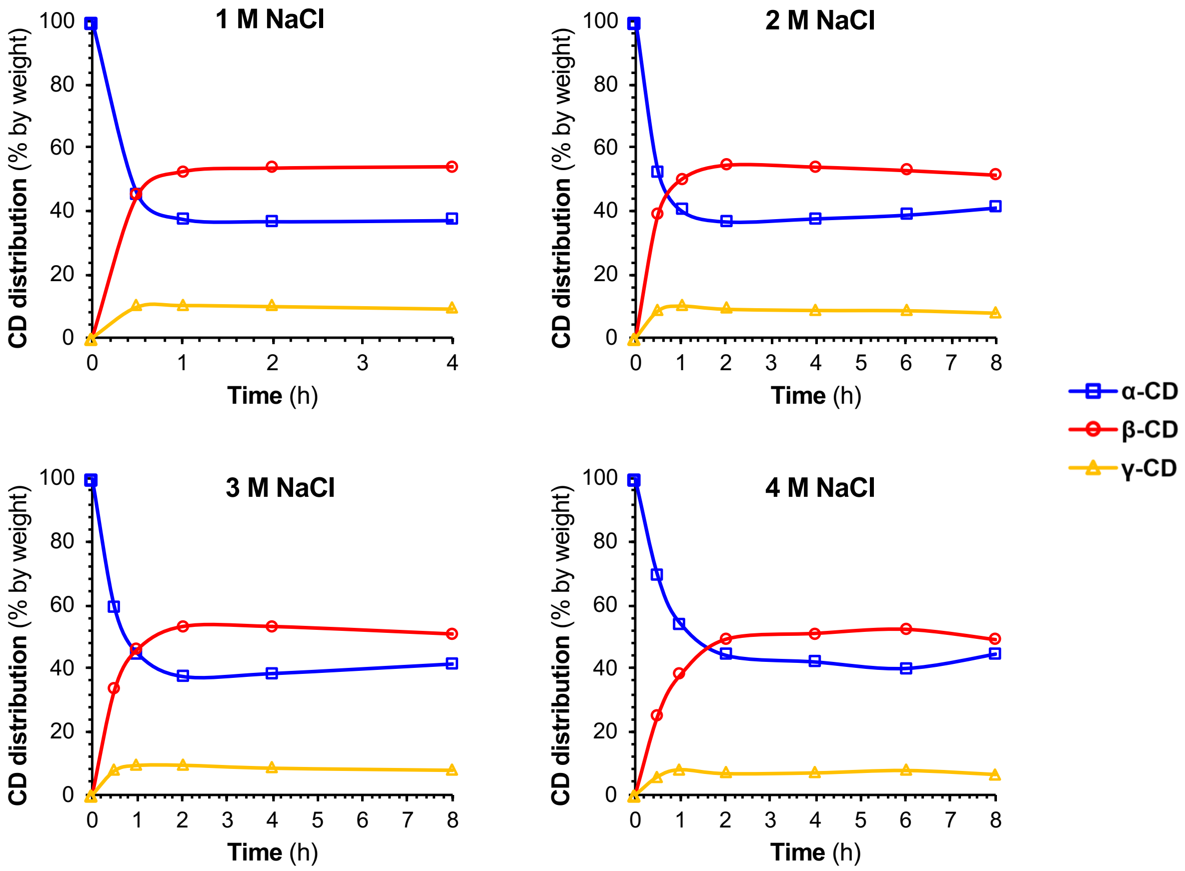

Supplementary Figure 2. Distribution of α-CD, β-CD and γ-CD as a function of time in reactions started from α-CD (10 mg/mL) in sodium phosphate buffer (50 mM, pH 7.5) with the indicated concentration of NaCl treated with CGTase at room temperature. (Lines connecting data points are only to guide the eye.)

1.3 $NaNO_3$

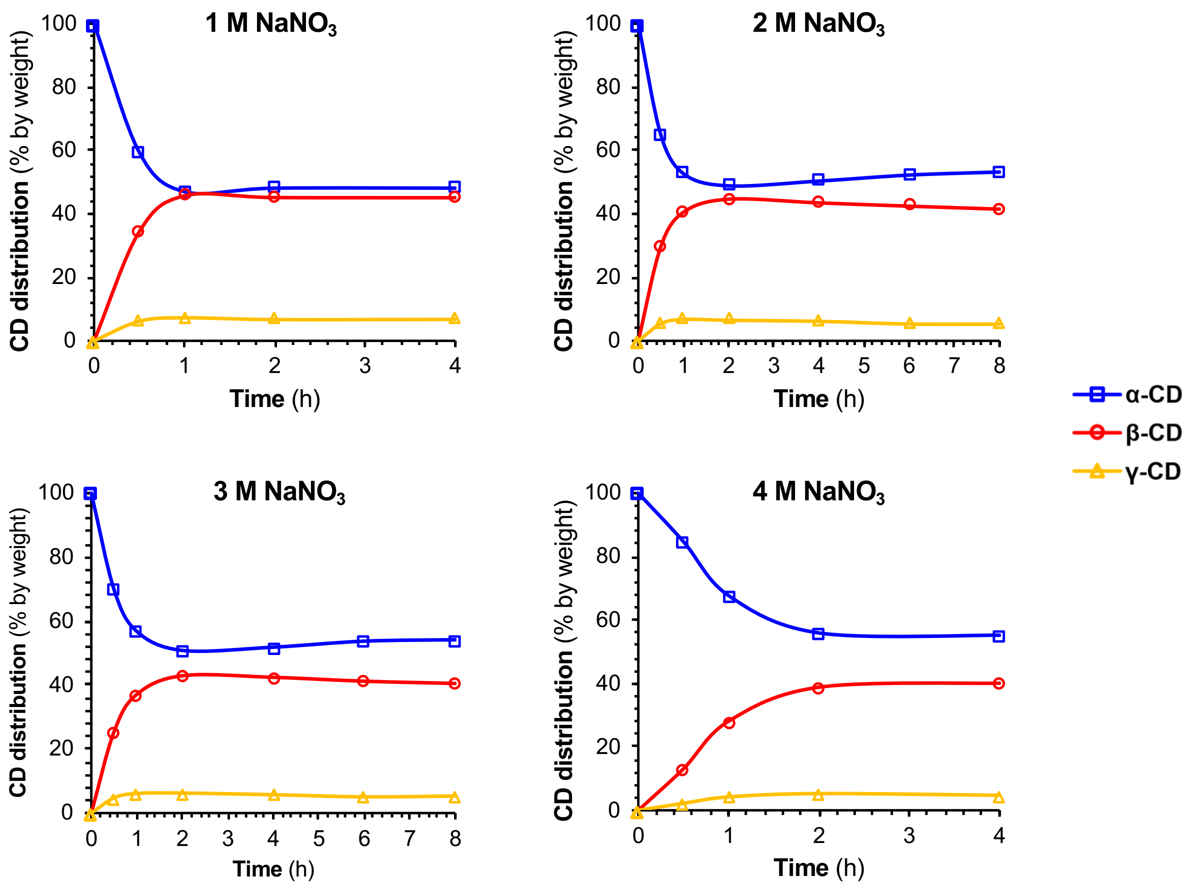

Supplementary Figure 3. Distribution of α-CD, β-CD and γ-CD as a function of time in reactions started from α-CD (10 mg/mL) in sodium phosphate buffer (50 mM, pH 7.5) with the indicated concentration of $NaNO_3$ treated with CGTase at room temperature. (Lines connecting data points are only to guide the eye.)

1.4 NaBr

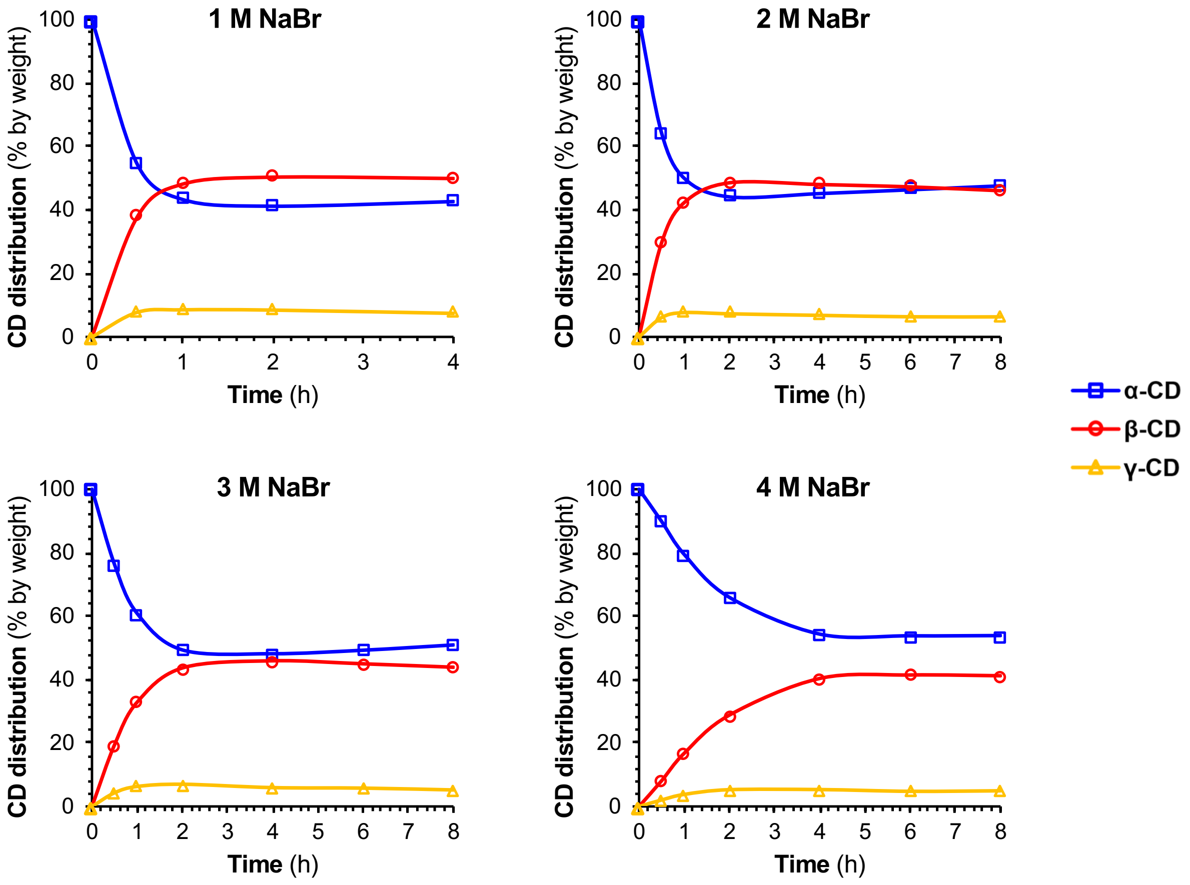

Supplementary Figure 4. Distribution of α-CD, β-CD and γ-CD as a function of time in reactions started from α-CD (10 mg/mL) in sodium phosphate buffer (50 mM, pH 7.5) with the indicated concentration of NaBr treated with CGTase at room temperature. (Lines connecting data points are only to guide the eye.)

1.5 $NaClO_4$

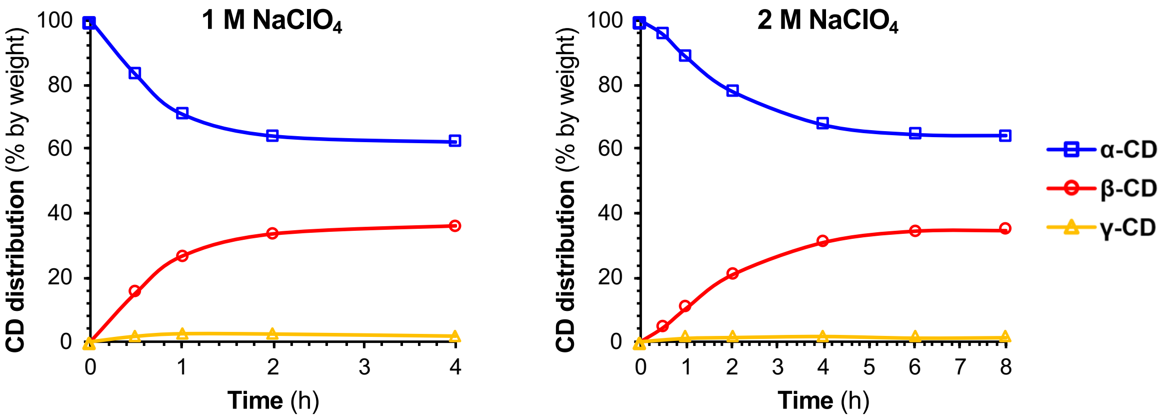

Supplementary Figure 5. Distribution of α-CD, β-CD and γ-CD as a function of time in reactions started from α-CD (10 mg/mL) in sodium phosphate buffer (50 mM, pH 7.5) with the indicated concentration of $NaClO_4$ treated with CGTase at room temperature. (Lines connecting data points are only to guide the eye.)

1.6 NaSCN

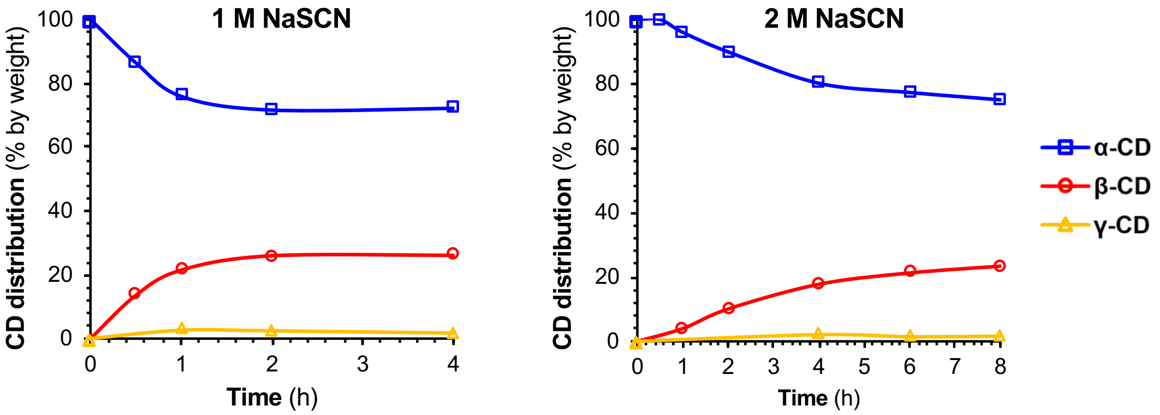

Supplementary Figure 6. Distribution of α-CD, β-CD and γ-CD as a function of time in reactions started from α-CD (10 mg/mL) in sodium phosphate buffer (50 mM, pH 7.5) with the indicated concentration of NaSCN treated with CGTase at room temperature. (Lines connecting data points are only to guide the eye.)

1.7 Enzymatic reactions with cyclohexanol as template with and without NaCl

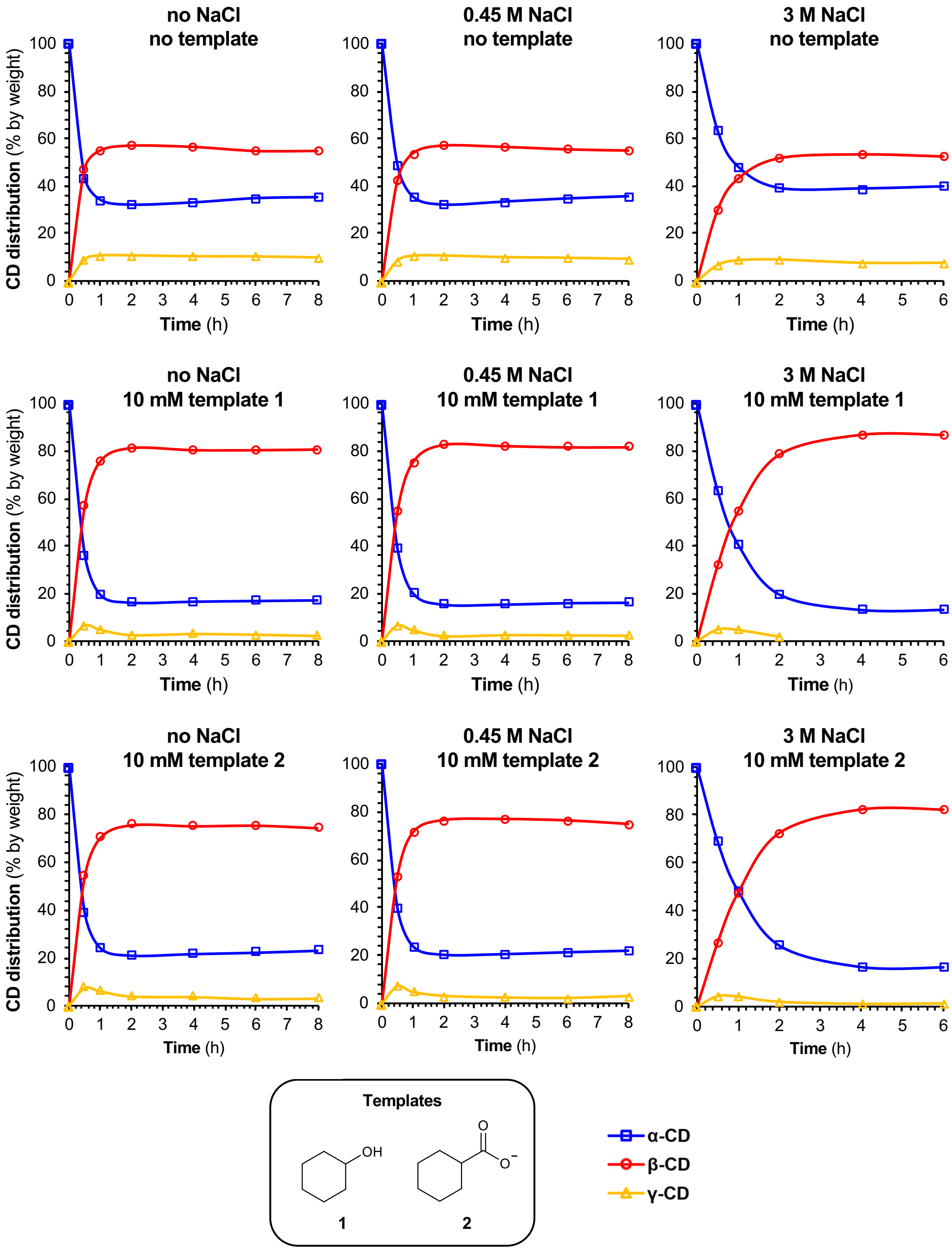

Supplementary Figure 7. Distribution of α-CD, β-CD and γ-CD as a function of time in reactions started from α-CD (10 mg/mL) and treated with CGTase at room temperature in sodium phosphate buffer (50 mM, pH 7.5) in the presence of the indicated concentrations of cyclohexanol, cyclohexane carboxylate and NaCl. (Lines connecting data points are only to guide the eye.)

2 Enzyme activity in the presence of denaturing salts NaSCN and $NaClO_4$

A series of solutions of CGTase in sodium phosphate buffer (50 mM, pH 7.5) with different sodium salts in concentrations up to 4 M were prepared. After 30 minutes of incubation of CGTase in salt solutions at room temperature, maltohexaose (**G6**) (10 mg/mL) was added. After 5 minutes, the reactions were quenched and the concentrations of **G6** remaining were determined by HPLC with an ELS detector. The consumption of **G6** relative to a reference (no salt added) are plotted in Supplementary Figure 8.

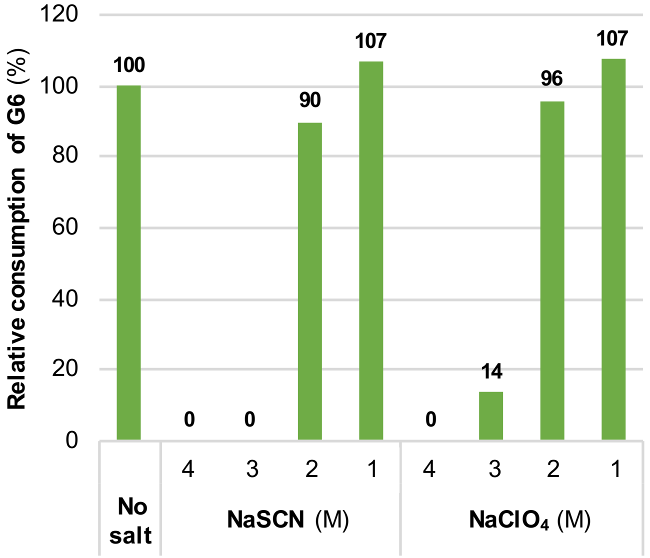

Supplementary Figure 8. Consumption of **G6** relative to a reference with no salt added after 30 minutes of exposing the enzyme to the salt solutions, followed by 5 minutes reaction time. With 3–4 M NaSCN a dramatic loss of enzyme activity was observed. With $NaClO_4$ NaSCN (1 M) and $NaClO_4$ (1 M) there was a perceived increase in activity, indicated by the increased consumption of **G6**. This is presumably due to the kinetic trapping of the α-CD / anion complex formed, leading to an apparent higher consumption of **G6** due to a slower rate of the reverse ring-opening reaction.

3 Simulations of dynamic combinatorial libraries

Simulations of dynamic combinatorial libraries using the program *DCLSim* requires the input of the relative formation constants K_f of the library members. K_f for α-, β-, and γ-CD were calculated according to Equation S1:

$$K_f(\text{CD-}n) = \frac{[\text{CD-n}]_{\text{eq}}}{[\text{G}_0]^{\text{n}}} \quad \textbf{(S1)}$$

Where CD-*n* is a cyclodextrin with a degree of polymerization *n*, K_f(CD-*n*) is the relative formation constant of the cyclodextrin, $[\text{CD-}n]_{eq}$ is the concentration (M) of the cyclodextrin at *pseudo*-equilibrium and $[G_0]$ is the total concentration of glucose units (M) in the cyclodextrin library at *pseudo*-equilibrium. The calculated K_f values are 9.5×10^4 M^{-5}, 2.6×10^6 M^{-6} and 7.6×10^6 M^{-7} for α-CD, β-CD, and γ-CD, respectively.
